# Atomic force microscopy investigation of the kinetic growth mechanisms of sputtered nanostructured Au film on mica: towards a nanoscale morphology control

**DOI:** 10.1186/1556-276X-6-112

**Published:** 2011-01-31

**Authors:** Francesco Ruffino, Vanna Torrisi, Giovanni Marletta, Maria Grazia Grimaldi

**Affiliations:** 1 Dipartimento di Fisica e Astronomia, Università di Catania via S. Sofia 64, 95123 Catania, Italy; 2 CNR-IMM MATIS, via S. Sofia 64, I-95123 Catania, Italy; 3 Laboratory for Molecular Surface and Nanotechnology (LAMSUN), Department of Chemical Sciences-University of Catania and CSGI, Viale A. Doria 6, 95125, Catania, Italy

## Abstract

The study of surface morphology of Au deposited on mica is crucial for the fabrication of flat Au films for applications in biological, electronic, and optical devices. The understanding of the growth mechanisms of Au on mica allows to tune the process parameters to obtain ultra-flat film as suitable platform for anchoring self-assembling monolayers, molecules, nanotubes, and nanoparticles. Furthermore, atomically flat Au substrates are ideal for imaging adsorbate layers using scanning probe microscopy techniques. The control of these mechanisms is a prerequisite for control of the film nano- and micro-structure to obtain materials with desired morphological properties. We report on an atomic force microscopy (AFM) study of the morphology evolution of Au film deposited on mica by room-temperature sputtering as a function of subsequent annealing processes. Starting from an Au continuous film on the mica substrate, the AFM technique allowed us to observe nucleation and growth of Au clusters when annealing process is performed in the 573-773 K temperature range and 900-3600 s time range. The evolution of the clusters size was quantified allowing us to evaluate the growth exponent 〈*z*〉 = 1.88 ± 0.06. Furthermore, we observed that the late stage of cluster growth is accompanied by the formation of circular depletion zones around the largest clusters. From the quantification of the evolution of the size of these zones, the Au surface diffusion coefficient was evaluated in $D\left(T\right)\;=\;[(\text{7}.\text{42}\;\times \;\text{1}0^{-\text{13}})\;\pm \;(\text{5}.\text{94}\;\times \;\text{1}0^{-\text{14}})\;{\text{m}}^{\text{2}}\text{/s}]\text{exp}\left(-\frac{(0.33\pm 0.04)\;\text{eV}}{kT}\right)$. These quantitative data and their correlation with existing theoretical models elucidate the kinetic growth mechanisms of the sputtered Au on mica. As a consequence we acquired a methodology to control the morphological characteristics of the Au film simply controlling the annealing temperature and time.

## Introduction

Thin nanometric films play important role in various fields of the modern material science and technology [1,2]. In particular, the structure and properties of thin metal films deposited on non-metal surfaces are of considerable interest [3,4] due to their potential applications in various electronic, magnetic and optical devices. The study of the morphology of such films with the variation of thickness and thermal processes gives an idea about the growth mechanism of these films [5-7]. Study of morphology and understanding of growth mechanism are, also, essential to fabricate nanostructured materials in a controlled way for desired properties. In fact, such systems are functional materials since their chemical and physical properties (catalytic, electronic, optical, mechanical, etc.) are strongly correlated to the structural ones (size, shape, crystallinity, etc.) [8]. As a consequence, the necessity to develop bottom-up procedures (in contrast to the traditional top-down scaling scheme) allowing the manipulation of the structural properties of these systems raised. Such studies find a renewed interest today for the potential nanotechnology applications [8]. The key point of such studies is the understanding of the thin film kinetic growth mechanisms to correlate the observed structural changes to the process parameters such as deposition features (i.e. rate, time, etc.) [9-13] and features of subsequent processes (i.e. annealing temperatures and time, ion or electron beam energy and fluence, etc.) [14-17].

In this framework, the study of the surface morphology of Au deposited on mica is crucial [18-39] in view of the fabrication of flat Au films for applications in biological, electronic, optical devices and techniques (i.e. surface enhanced Raman spectroscopy). Mica is a suitable support for crystalline Au deposition because the small mismatch of the crystal lattice allows the Au to grow in large atomically flat areas. The understanding of the kinetic growth mechanisms of Au on mica allows to tune the process parameters (substrate temperature, pressure, rate deposition, film thickness) to obtain ultra-flat Au film as suitable platform for anchoring self-assembling monolayers (due to Au affinity to thiol groups of organic molecules), molecules, nanotubes, nanoparticles and so on. Atomically flat Au substrates are ideal for imaging adsorbate layers using scanning probe microscopy techniques. For these characterization methods, flat substrates are essential to distinguish the adsorbed layer from the substrate features. Obviously, the control of the kinetic growth mechanisms of Au on mica is a prerequisite for control of the film nano- and micro-structure to obtain materials with desired morphological properties. The main literature concerns Au film on mica produced by ultra-high-vacuum evaporation [18-25,29-34,37-39]. Very few works regard sputtered Au films on mica [22,26-28] and the general deposition criteria deduced for the evaporation technique do not necessarily apply to other methods. The sputtering method is simpler than vacuum evaporation both for instrumentation and deposition procedure; with the deposition parameters properly chosen, the sputtered films exhibit superior surface planarity, even flatter than the smoothest evaporated films reported to date [28].

In the present work we aim to illustrate the surface morphology evolution of room-temperature sputtered nanoscale Au film on mica when it is subjected to annealing processes. We deposited 28 nm of Au on the mica substrate and performed annealing treatments in the 573-773 K temperature range and 900-3600 s time range to induce a controlled film nano-structuring.

Atomic force microscopy (AFM) is an important methodology to study the surface morphology in real space [40,41]: the top surface can be imaged using an AFM and these images provide information about the morphology evolution. So, using the AFM technique, we analyzed quantitatively the evolution of the Au film morphology as a function of the annealing time and temperature. Such a study allowed us to observe some features of the morphology evolution and to identify the film evolution mechanisms. In particular, several results were obtained:

1. In a first stage of annealing (573 K-900 s) a nucleation process of small clusters from the starting quasi-continuous 28 nm Au film occurs.

2. In a second stage of annealing (573-773 K for 1800-3600 s) a growth process of the Au clusters occurs. The late state of cluster growth is accompanied by the formation of circular depletion zones around the largest clusters. This behavior was associated, by the Sigsbee theory [42], to a surface diffusion-limited Ostwald ripening growth in which the Au surface diffusion plays a key role.

3. The AFM analyses allowed to study the evolution of the mean cluster height as a function of annealing time for each fixed temperature, showing a power-law behavior characterized by a temporal exponent whose value suggest that the full cluster surface is active in mass transport.

4. By the evolution of the mean radius of the depletion zones as a function of the annealing time t and temperature *T *the Au surface diffusion coefficient at 573, 673, and 773 K was estimated.

5. The activated behavior of the Au surface diffusion coefficient was studied obtaining the activation energy for the surface diffusion process.

## Experimental

Samples were prepared from freshly cleaved mica substrates. Depositions were carried out by a RF (60 Hz) Emitech K550x Sputter coater onto the mica slides and clamped against the cathode located straight opposite of the Au source (99.999% purity target). The electrodes were laid at a distance of 40 mm under Ar flow keeping a pressure of 0.02 mbar in the chamber. The deposition time was fixed in 60 s with working current of 50 mA. In these conditions, the rate deposition was evaluated in 0.47 nm/s and, accordingly, the thickness *h *of the deposited film was about 28 nm.

The annealing processes were performed using a standard Carbolite horizontal furnace in dry N_2 _in the 573-773 K temperature range and 0-3600 s time range.

The AFM analyses were performed using a Veeco-Innova microscope operating in high amplitude mode and ultra sharpened Si tips were used (MSNL-10 from Veeco Instruments, with anisotropic geometry, radius of curvature approximately 2 nm, tip height approximately 2.5 μm, front angle approximately 15°, back angle approximately 25°, side angle 22.5°) and substituted as soon as a resolution lose was observed during the acquisition. The AFM images were analyzed by using the SPMLabAnalyses V7.00 software.

Rutherford backscattering spectrometry (RBS) analyses performed using 2 MeV ^4^He^+ ^backscattered ions at 165°.

## Results

**Figure 1:**
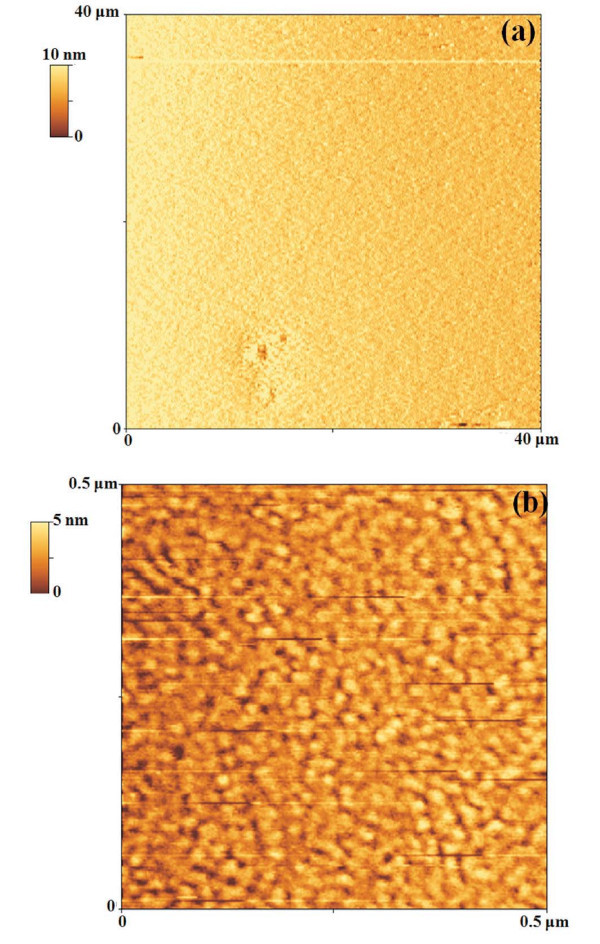
AFM images of the starting Au film: (a) 40 μm × 40 μm AFM scan of the starting 28-nm Au film sputter-deposited on the mica substrate; (b) 0.5 μm × 0.5 μm AFM scan of the same sample, to evidence the percolative nature of the film.

Figure 1a shows a 40 μm × 40 μm AFM image of the starting 28 nm Au film. We can observe that over such a scan size the Au film is very flat presenting a roughness *σ *= 1.2 nm. The roughness was evaluated using the SPMLabAnalyses V7.00 software: it is defined by $\sigma ={\left[\frac{1}{N}\sum_{i=1}^{N}{\left(y_{i}-\bar{y}\right)}^{2}\right]}^{1/2}$ where *N *is the number of data points of the profile, *y*_*i *_are the data points that describe the relative vertical height of the surface, and $\bar{y}$ is the mean height of the surface. Furthermore, the roughness value was obtained averaging the values obtained over three different images.

Figure 1b shows a 0.5 μm × 0.5 μm AFM image of the starting 28 nm Au film, to highlight its nanoscale structure: we can observe the occurrence of a percolation morphology (Au islands grow longer and are connected to form a quasi-continuous network across the surface) as standard for metal film on non-metal surface in the late stage of growth [12,43-45]. In fact, generally, metal films on non-metal surfaces grow in a first stage (low thicknesses) in the Volmer-Weber mode as 3D islands with droplet-like shapes. For higher thicknesses, the shapes of the islands become elongated (and, correspondently, their surface density decreases), and only for further higher thicknesses the film takes a percolation morphology and finally becomes a continuous rough film.

**Figure 2:**
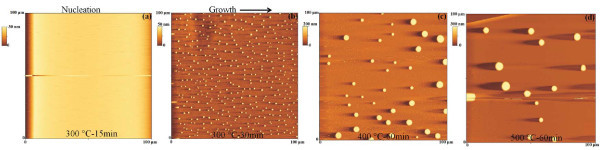
**100 μm × 100 μm AFM scans of the Au film thermally processed at: (a) 573 K-15 min, (b) 573 K-30 min, (c) 673 K-60 min, and (d) 773 K-60 min**.

We studied the evolution of the starting ultra-flat 28 nm sputter-deposited Au film as a consequence of the annealing processes performed in the 573-773 K temperature range and 0-3600 s time range. So, as examples, Figure 2 reports 100 μm × 100 μm AFM images of the starting Au film subjected to various thermal treatments: (a) 573 K-900 s, (b) 573 K-1800 s, (c) 673 K-3600 s, and (d) 773 K-3600 s. In particular, the AFM image in Figure 2b of the sample annealed at 573 K-1800 s shows the formation of Au clusters whose size increases when the annealing time and/or temperature increases, while their surface density (number of clusters per unit area) decreases.

**Figure 3:**
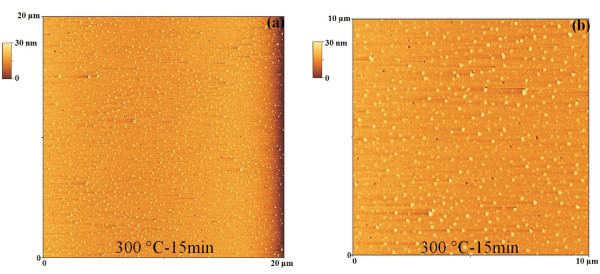
AFM images of the thermally processed Au film: (a, b) 20 μm × 20 μm and 10 μm × 10 μm, respectively, AFM scans of the Au film thermally processed at 573 K-15 min.

**Figure 4:**
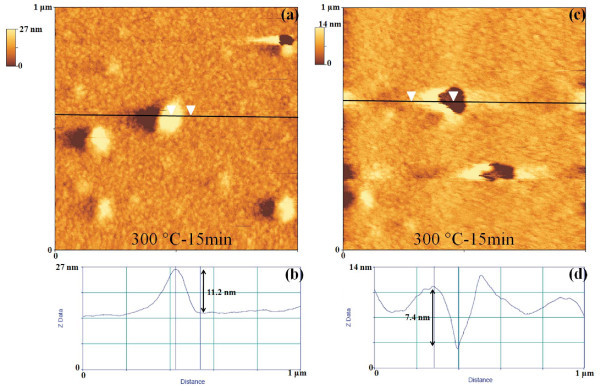
AFM images and section masurements of the thermally processed Au film: (a, c) 1 μm × 1 μm AFM scans of the Au film thermally processed at 573 K-15 min; (b) section measurement to estimate the height (11.2 nm) of a nucleated Au cluster; (d) section measurement to estimate the depth (7.4 nm) of a hole in the Au film.

**Figure 5:**
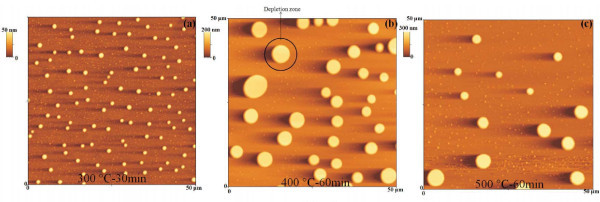
**50 μm × 50 μm AFM scans of the Au film thermally processed at: (a) 573 K-30 min, (b) 673 K-60 min, and (c) 773 K-60 min**.

**Figure 6:**
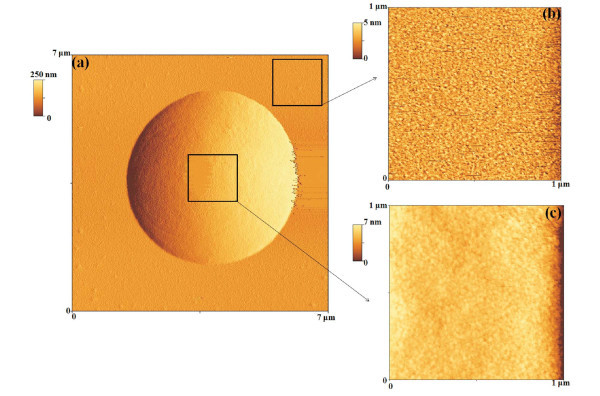
AFM image of a single Au cluster: (a) 7 μm × 7 μm AFM scan of the Au film thermally processed at 773 K-60 min, focusing, in particular, on an Au cluster; (b) 1 μm × 1 μm AFM scan of the underlaying Au film; (c) 1 μm × 1 μm AFM scan on the Au cluster, evidencing its granular structure.

To understand the formation of the Au clusters, first of all, we analyzed the morphology of the starting Au film after the 573 K-900 s. So, Figure 3a,b shows 20 μm × 20 μm and 10 μm × 10 μm AFM images of the Au film annealed at 573 K-900 s. Interestingly, we observe that this annealing process determines the nucleation of small Au clusters (height of about 10 nm) from the starting quasi-continuous film. Furthermore, while the nucleation of these small clusters takes place, also the formation of small holes (depth of about 10 nm) in the Au film occurs. Figure 4 reports, also, 1 μm × 1 μm AFM images of the same sample focusing both on the small Au clusters and the holes. Figure 4b shows an AFM cross-sectional line scanning profile analysis that refers to a Au cluster imaged in Figure 4a: the section analyses allow to evaluate its height in 11.2 nm. Similarly, Figure 4d shows the AFM cross-sectional line scanning profile analysis that refers to an hole imaged in Figure 4c, allowing to evaluate its depth in 7.4 nm. We can conclude that the 573 K-900 s annealing process determines the first stage of nucleation of Au clusters from the starting quasi-continuous film and that the following annealing processes cause their growth. To study the growth stage, we imaged by the AFM the Au clusters annealed between 573 and 773 K and 0-3600 s at higher resolution. As examples, Figure 5 reports 50 μm × 50 μm AFM images of the starting Au film subjected to various thermal treatments: (a) 573 K-1800 s, (b) 673 K-3600 s, and (c) 773 K-3600 s. The qualitative increase of the mean clusters size and the decrease of their surface density increasing the annealing time *t *and/or temperature *T *are evident. The main feature in the late stage of the cluster growth is the formation of circular depletion zones around the largest clusters. We used the AFM analyses, also, to image the morphology structure of the large clusters and of the depletion zones around them. So, for examples, Figure 6a shows a 7 μm × 7 μm AFM image of a single Au large cluster (corresponding to the 673 K-3600 s annealed sample), while Figure 6b shows a 1 μm × 1 μm AFM image of depletion zone near the cluster, and Figure 6c shows a 1 μm × 1 μm AFM image taken over the Au cluster. Figure 6b shows a percolation morphology of the underlaying residual Au film (similar to that of the starting 28 nm Au film), while Figure 6c shows a more complex nano-structure: the large cluster appears to be formed by Au nanoclusters.

## Discussion

**Figure 7:**
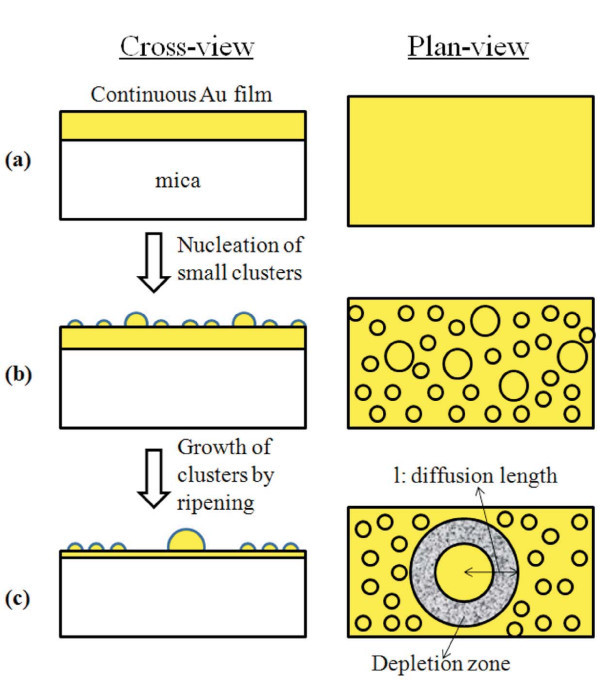
**Schematic picture of the growth stages of the Au film as a function of the thermal budget**.

On the basis of the exposed results, we can sketch the evolution of the Au film morphology as pictured in Figure 7: starting from the quasi-continuous Au film (Figure 7a), the 573 K-900 s annealing process determines the first stage of nucleation of Au clusters from the starting quasi-continuous film (Figure 7b). After the nucleation stage, the subsequent annealing in the 573-773 K temperature range and 0-3600 s time range determines a growth stage of the nucleated clusters with the formation of depletion zones around the largest clusters (Figure 7c). In particular, this phenomenon corresponds to the surface diffusion-limited Ostwald ripening model developed by Sigsbee [42]. Ostwald ripening is regulated by the vapor pressure at the surfaces of the cluster, *P*(*R*), depending on the curvature of the surface and it is driven by the minimization of the total surface free energy. For spherical clusters with a radius *R*, the vapor pressure at the surface of the cluster is given by the following relation according to the Gibbs-Thompson equation [46]:

(1) $$P(R)=P_{\infty }\exp(2\gamma \Omega /Rk_{\text{B}}T)\approx P_{\infty }(1+c/R)$$

with *P*_∞ _the vapor pressure at a planar surface, *γ *the surface free energy, *Ω *is the atomic volume, *k*_B _the Boltzmann constant, *c *a temperature-dependent but time-independent constant and depending on the surface diffusion atomic coefficient *D*_S _[46-48]. Lifshitz and Slyozow [46] as well Wagner [47] have formulated the basis for a mathematical description of the growth of grains in three-dimensional systems, yielding the following general expression for the asymptotic temporal evolution mean particle radius 〈*R*〉

(2) $$\langle R\rangle \approx \;ct^{\text{1}/z}$$

*z *being a characteristic growth exponent whose value depends on the specific characteristics of the growth mechanism. At any stage during ripening there is a so-called critical particle radius *R*_c_: particles with *R *>*R*_c _will grow and particles with *R *<*R*_c _will shrink. The atoms of the clusters with *R *<*R*_c _diffuse over the surface toward the nearest cluster with *R *>*R*_c _and they are incorporated by it. Later, Sigsbee [42] developed a model for the cluster growth in two dimensions and considered the formation of depletion zones. A depletion zone around a large cluster, originates from the shrunken smaller clusters. Such depletion zones would have circular border lines in the case of the clusters being generated on isotropic smooth substrates, that is if the diffusion process occur isotropically. The radius l of a depletion zone at time t is simply the atomic diffusion length:

(3) $$l=\sqrt{D_{\text{s}}t}.$$

The time dependence of the cluster growth expressed by Equation 2 is determined by the dimensionality of the growing system and the processes limiting the mass transport by surface diffusion. The specific values of *z *for different systems are summarized in [7]. For example, for the three-dimensional cluster growth with only the contact line to the substrate surface active in mass transport, the critical radius of the clusters will grow according to Equation 2 with a time exponent 1/*z *= 1/3; if, instead, for the three-dimensional clusters the full cluster surface is active in mass transport, a time exponent 1/*z *= 1/2 is expected.

Obviously, the mass conservation law dictates that increasing 〈*R*〉 the thickness of the underlaying quasi-continuous film has to decreases proportionally, as qualitatively indicated by the schematic picture in Figure 7.

**Figure 8:**
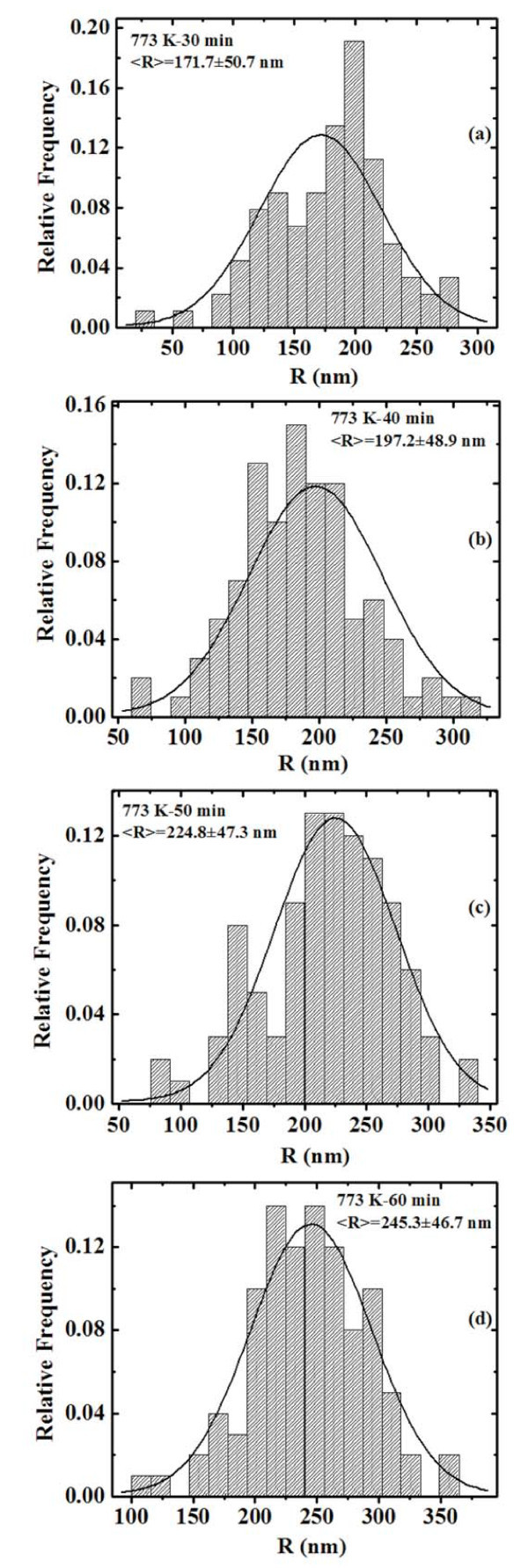
**Distributions of the clusters height *R *for samples annealed at 773 K for: (a) 30 min, (b) 40 min, (c) 50 min, and (d) 60 min.** The continuous lines are the Gaussian fits.

**Figure 9:**
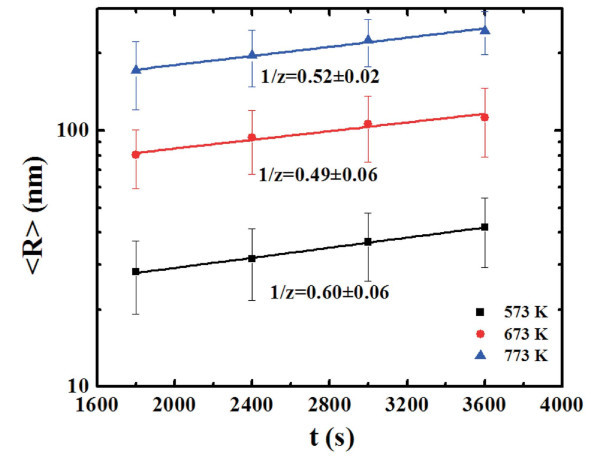
**Plot (dots) of the mean clusters height, 〈*R*〉, as a function of the annealing time *t*, for each fixed annealing temperature *T***. The continuous lines are the fits.

We can quantify the evolution of the height *R *of the clusters by the AFM analyses using the SPMLabAnalyses V7.00 software that define each grain area by the surface image sectioning of a plane that was positioned at half grain height. In this way we can obtain the distributions of *R *as a function of the annealing time *t *for each fixed annealing temperature *T*. Figure 8 reports, for examples, the distributions of *R *for the samples annealed at 773 K-1800 s (a), 773 K-2400 s (b), 773 K-3000 s (c), and 773 K-3600 s (d), respectively. Each distribution was calculated on a statistical population of 100 grains and fitted (continuous lines in Figure 8) by a Gaussian function whose peak position was taken as the mean value 〈*R*〉 and whose full width at half maximum as the deviation on such value. Therefore, we obtain the evolution of the mean clusters height 〈*R*〉 as a function of *t *for each fixed *T*, as reported in Figure 9 (dots) in a semi-log scale. For each temperature we fitted (continuous lines in Figure 9) the experimental points by Equation 2 to obtain the best value for 1/*z*: by this procedure we obtain 1/*z *= 0.52 ± 0.02 at 573 K, 1/*z *= 0.49 ± 0.06 at 673 K, and 1/*z *= 0.60 ± 0.06 at 773 K. Averaging these values we deduce 1/*z *= 0.54 ± 0.04 indicating a three-dimensional cluster growth in which the full clusters surface is active in the mass transport.

**Figure 10:**
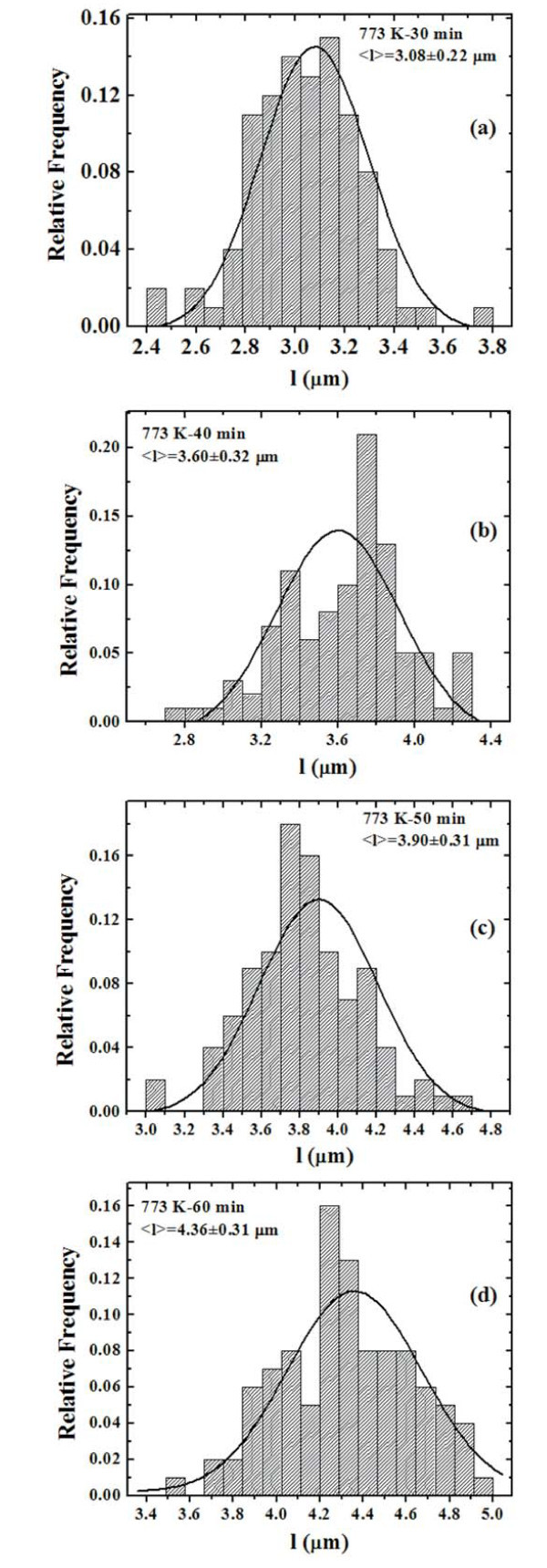
**Distributions of the radius l of the depletion zones for samples annealed at 773 K for: (a) 30 min, (b) 40 min, (c) 50 min, and (d) 60 min**. The continuous lines are the Gaussian fits.

**Figure 11:**
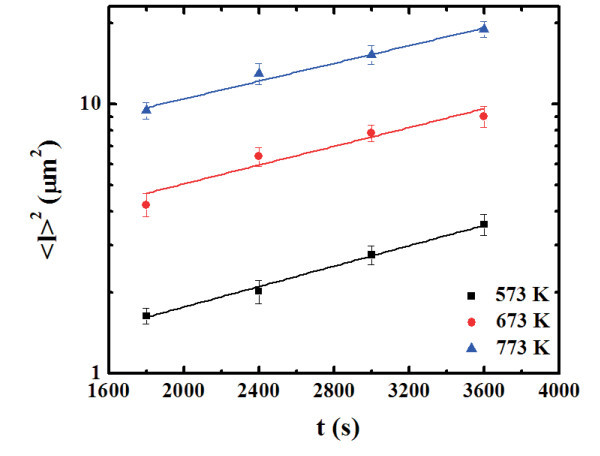
**Plot (dots), in semi-log scale, of the square values of the mean radius of the depletion zones, 〈*l*〉**^**2**^**, as a function of the annealing time *t*, for each fixed annealing temperature *T***. The continuous lines are the fits.

**Figure 12:**
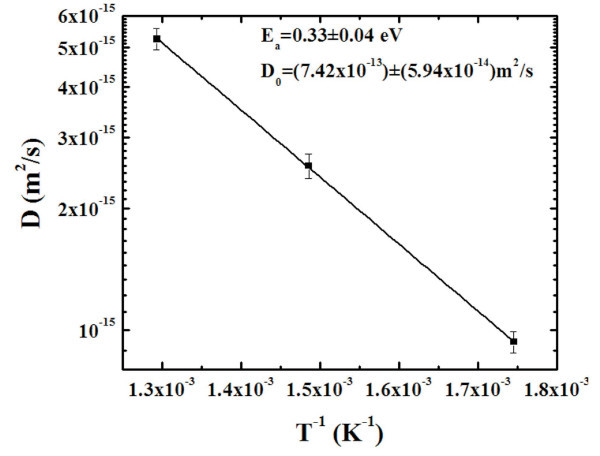
**Plot (dots), in semi-log scale, of the Au surface diffusion coefficient as a function of the inverse of the temperature.** The continuous line is the fit.

By the AFM analyses we can, also, quantify the evolution of the radius *l *of the depletion zones observable in the AFM images around the larger clusters. Also in this case we can proceed to a statistical evaluation of 〈*l*〉: by the analyses of the AFM images we obtain the distributions of *l *as a function of the annealing time t for each fixed annealing temperature *T*. Figure 10 reports, for examples, the distributions of *l *for the samples annealed at 773 K-1800 s (a), 773 K-2400 s (b), 773 K-3000 s (c), and 773 K-3600 s (d), respectively. Each distribution was calculated on a statistical population of 100 grains and fitted (continuous lines in Figure 10) by a Gaussian function whose peak position was taken as the mean value 〈*l*〉 and whose full width at half maximum as the deviation on such value. Therefore, we obtain the evolution of the mean clusters height 〈*l*〉 as a function of *t *for each fixed *T*. In Figure 11, we plot (dots) in a semi-log scale 〈*l*〉^2 ^as a function of *t *for each *T*, obtaining linear relations as prescribed by Equation 3. Fitting the experimental data by 〈*l*〉^2 ^= *D*_s_t we obtain, as fit parameter, the values of the atomic Au surface diffusion coefficient *D*_S_: *D*_S_(573 K) = (9.35 × 10^-16^) ± (5.6 × 10^-17^) m^2^/s, *D*_S_(673 K) = (2.55 × 10^-15^) ± (1.8 × 10^-16^) m^2^/s, *D*_S_(773 K) = (5.25 × 10^-15^) ± (3.2 × 10^-16^) m^2^/s. The Arrhenius plot of the resulting *D*_s_(*T*), showen in Figure 12 indicates the occurrence of the thermally activated diffusion process [6,49] described by

(4) $$D_{\text{s}}(T)=D_{0}e^{-\frac{E_{\text{a}}}{k_{B}T}}$$

*D*_0 _being the pre-exponential factor and *E*_a _the activation energy of the surface diffusion process. By the fit of the experimental data (dots) in Figure 12 using Equation 4 we obtain, as fit parameters, *D*_0 _= (7.42 × 10^-13 ^± 5.9 × 10^-14^) m^2^/s and *E*_a _= (0.33 ± 0.04)eV/atom.

A consistency calculation is suggested by the mass conservation law: at any stage of annealing process the total amount of deposited Au must be constant. By the RBS analyses, the starting 28 nm Au film was found to be formed by *Q *= 1.7 × 10^17 ^atoms/cm^2^. After, for example, the final 773 K-3600 s annealing process, the total amount of the Au atoms forming the Au cluster and the underlaying residual quasi-continuous film must be the same. If we suppose the largest Au clusters obtained after the 773 K-3600 s annealing as semi-spheres of radius 〈*R*〉 = 240 nm with a surface density, estimated by the AFM images of about *N *= 9 clusters per 100 μm^2^, then the number *S *= *N*(4/6)〈*R*〉^3^/Ω ≈ 1.5 × 10^17 ^atoms/cm^2 ^is an estimation of the Au atoms per unit area forming these Au clusters. The remaining (1.7 × 10^17^-1.5 × 10^17^) Au/cm^2 ^= 2 × 10^16 ^Au/cm^2 ^form the underlaying residual Au film. This amount corresponds to an average thickness of about 3 nm. This calculation gives a reasonable confirmation of the mass conservation law validity.

Concerning the formation of the small holes in the Au film, as evidenced in the AFM images in Figures 3 and 4, as already done in [13], we can suppose that the formation of this holes is characteristic of the sputtering deposition technique. In fact, it is known from the literature that when Au films on mica are bombarded with noble gas ions at low energies [22,28,50-52] (as in the case of Au film surface processed by RF Ar plasma [50]) stable surface defects (holes) with a monoatomic layer depth are produced. For example, when Au(111) films on mica were bombarded with helium ions at energies of 0.6 or 3 keV, holes with a monoatomic layer depth were observed using STM [52]. Their formation is due to the clustering of vacancies produced by individual sputtering events. Furthermore, for an initially atomically flat Au surface on mica, the flat surface features were observed to be modified during 3 keV Ar irradiation by the ablation of small clusters of atoms which then diffused until a sputter-etched pit was encountered, in which they were trapped [22]. It has been suggested [22], also, that the high energetic sputtered atoms (in comparison with evaporated atoms) from the target with their energetic impact with the growing film surface would cause a poorly oriented pebble-like structure for Au films sputtered onto a RT mica. In our experimental conditions, the Ar^+ ^ions have energy of 0.23 keV, whereas the sputtering threshold for Ar^+ ^ions on Au is about 20 eV, and at 0.23 keV, 1 Au atom is sputtered for each Ar^+ ^ions [53]. On the basis of such considerations we can suppose that during the sputter deposition of the starting 28 nm Au film, stable surface defects with a monoatomic layer depth are produced by the interaction of the Ar plasma with the growing Au film. The subsequent annealing processes induce a coalescence phenomenon of these defects resulting in the formation of the observed holes.

## Conclusions

AFM has been applied for the analysis of the dynamics morphology evolution of room-temperature sputtered Au film on mica. In particular, an analysis of the structural evolution of a starting 28-nm Au film as a consequence of annealing processes was performed. The nucleation and growth of Au cluster, as a consequence of the thermal treatments were observed and the possibility of controlling their size by process parameters such as annealing time and/or temperature has been demonstrated, describing their kinetic growth mechanism. In particular, the clustering kinetic process has been interpreted by classical models involving surface diffusion-limited ripening of three-dimensional clusters on a substrate. From the quantification of the time evolution of the mean cluster height, a time exponent 1/*z *= 0.54 ± 0.04 was evaluated, indicating a three-dimensional cluster growth in which the full clusters surface is active in the mass transport. Furthermore, from the observation of the formation of depletion zones around the largest clusters and by the quantification of their time evolution, the Au surface diffusion coefficient *D*_s_(*T*) = (7.42 × 10^-13 ^± 5.9 × 10^-14^)exp[(0.33 ± 0.04)eV/*k*_*B*_*T*]m^2^/s was evaluated.

The results of the present work can be of importance in view of the tuning of the morphological characteristics of the sputter-deposited Au films on mica for various technological applications as anchoring of molecules and nanotubes, optoelectronic and plasmonic devices, etc. About analysis techniques, the nano- and micro-structured Au films on mica presented in this work could be of interest, for example, for surface enhanced Raman spectroscopy (SERS) and surface resonance plasmonic (SPR) applications as plasmonic substrates.

## Abbreviations

AFM: atomic force microscopy; RBS: Rutherford backscattering spectrometry; SERS: surface enhanced Raman spectroscopy; SPR: surface resonance plasmonic.

## Competing interests

The authors declare that they have no competing interests.

## Authors' contributions

FR conceived the study, and participated in its design and coordination; performed the gold sputter deposition, the annealing processes and the atomic force microscopy analyses; developed the theoretical framework for the analyses of the experimental data; analyzed the experimental data; drafted the manuscript.

VT conceived the study, and participated in its design; supplied and prepared the mica substrates; participated in the development of the theoretical framework for the analyses of the experimental data; contributed in drafting the manuscript.

GM: conceived the study, and participated in its design; participated in the development of the theoretical framework for the analyses of the experimental data; contributed in drafting the manuscript.

MGG: conceived the study, and participated in its design and coordination; participated in the development of the theoretical framework for the analyses of the experimental data; contributed in drafting the manuscript.

All authors read and approved the final manuscript.
